# Annual Meeting of the Alumni Association, Medical Department of Niagara University

**Published:** 1888-05

**Authors:** 


### *ANNUAL MEETING OF THE ALUMNI ASSOCIATION, MEDICAL DEPARTMENT OF NIAGARA UNIVERSITY.*

Convocation day brought together many of the sons of "Niagara," and no little interest was taken in the proceedings of the association by the medical men of the city. The morning meeting was devoted to business matters and the election of officers, which resulted as follows: President, Dr. C. C. Frederick; first vice-president, Dr. E. J. Murphy; second vice-president, Dr. L. J. Hanley; secretary, Dr. C. G. Congdon; treasurer, Dr. H. D. Ingraham; executive committee, Drs. George W. T. Lewis, F. R. Campbell and F. H. Potter.

The address of welcome, on the part of the faculty, was made by Prof. Thomas Lothrop. He said:

Gentlemen—On this auspicious occasion, which calls together annually, from widely distant sections, the alumni and friends of the medical department of Niagara University, the faculty bids me welcome her sons.

Every movement which enters, as a factor, into the solution of the great problem of human progress, must be inspired by certain important fundamental principles. The medical profession have felt the influence of public sentiment for higher medical education. This sentiment has been fully recognized here as elsewhere. Medicine, as a profession, has too often been degraded to the position of a trade, offering to scheming adventurers and mercenary charlatans a fertile field for the exercise of their impious gifts. Medical schools, conducted on business principles, with a view to contribute money to covetous professors rather than to benefit the human race through the advancement of knowledge, have been willing instruments in this work of debasement. The recognition of these facts, so fully appreciated by the better element in the profession, led to the establishment of this medical school. The trustees of the university selected men to carry into effect their object, in sympathy with this higher sentiment of the profession and the public. The movement, thus inaugurated, has brought with it a certain responsibility, which is felt by the medical faculty of the university, and must be shared by the alumni, upon whom we depend for encouragement and material support. It matters not how meritorious may be our motives or how high our aims, associated effort, inspired by strong convictions of duty and of right, and encouraged by a common sympathy and a common purpose, is absolutely necessary for the maintenance and advancement of so great a movement. These facts demonstrate forcibly the necessity of such an organization as the Associated Alumni to give moral support to the faculty in their responsible labors.

Fully realizing the magnitude of the work before them, the trustees incorporated into the organic law of the university a compulsory three years' course of instruction, which is possessed by no other medical college in this state, and also the divorcement of the teaching and the licensing powers, so that every candidate for the degree of doctor of medicine, must submit his professional attainments to a critical examination before the board of examiners, composed of distinguished members of the profession, and called to their work independently of the teaching power. A further restriction is also made, by requiring a certain standard of intellectual attainments of all students who apply for admission into the school. These features, incorporated into the charter of the University, have been advocated by the best minds in the profession. In five years, receiving a generous support from the parent university, and encouraged by a strong sentiment in the profession in favor of a higher medical education, the medical faculty have established a school of medicine upon the fundamental principles above enunciated, have presented, in this period, two classes for the degree of Doctor of Medicine, of splendidly equipped men, and to-day send before the board of examiners twelve men, good and true, who have successfully stood the test of a written examination, occupying an entire week, which, in the wide range of subjects and severity, cannot be exceeded by an institution in this country.

Speaking in behalf of my colleagues, we depend upon the alumni for support in this important work. We do not ask your sympathy and aid for our personal and professional aggrandizement. We have at heart the important work, undertaken by the university, of thoroughly educating medical men *pro bono publico.*

Medicine, however, is a progressive science. It needs earnest workers and zealous students. Original research and investigation can be prosecuted here in our laboratories, as well as elsewhere. Original thinkers and indefatigable laborers are not wanting in this new country, so absorbed in mercenary pursuits. We instance the important introduction to the profession, of "forced respiration," by George E. Fell, of our faculty, in opium narcosis and asphyxia, as an earnest of what every member of our faculty endeavors to do for the profession and the public. We need material aid to support men in their investigations of disease. Among our money princes in this wealthy city, is there not one who will endow a professorship or found a laboratory in this college, which will enable us to prosecute scientific work, original investigation into the unknown of disease, which will bless in its results the human race?

Almost single-handed and alone, and antagonized by an unscrupulous opposition, we have accomplished more than our most sanguine anticipations. In the future, aided by the efforts of the alumni and encouraged by the better element in the profession, and materially assisted by the wealth and intelligence of our city, we hope to become, at a day not far distant and in a fuller measure than at present, a beacon light which will send its beneficent rays far and wide over our beloved country and over the world, to alleviate human suffering and to bless mankind.

The afternoon session was devoted entirely to the reading and discussion of papers, and we are glad to be able to announce that in subsequent numbers of the JOURNAL we will present to our readers the very admirable papers which were, at this meeting, read before the association.

